# Composition and Ecological Functionality of Fungal Communities Associated with Smokeless Tobacco Products Mainly Consumed in India

**DOI:** 10.1128/spectrum.02273-21

**Published:** 2022-06-13

**Authors:** Mohammad Sajid, Sonal Srivastava, Ravi Kumar Yadav, Harpreet Singh, Shalini Singh, Mausumi Bharadwaj

**Affiliations:** a Division of Molecular Genetics and Biochemistry, Molecular Biology Group, ICMR-National Institute of Cancer Prevention and Research, Noida, Uttar Pradesh, India; b Division of Biomedical Informatics, Indian Council of Medical Research (ICMR), New Delhi, India; c ICMR-National Institute of Cancer Prevention and Research, Noida, Uttar Pradesh, India; d WHO-FCTC Global Knowledge Hub on Smokeless Tobacco, ICMR-National Institute of Cancer Prevention and Research, Noida, Uttar Pradesh, India; University of Mississippi

**Keywords:** FUNGuild, mycobiome, mycotoxins, oral cancer, smokeless tobacco products, tobacco-specific nitrosamines

## Abstract

The microbial communities present in smokeless tobacco products (STPs) perform critical steps in the synthesis of carcinogens, mainly tobacco-specific nitrosamines (TSNAs). Most studies emphasize the bacterial component, and the mycobiome of STPs has not been well characterized. In this study, we investigated the fungal communities in the different categories of STPs by sequencing the internal transcribed spacer (ITS) rRNA region of the fungal genome. The ecological character of the fungal community associated with STPs was determined by using FUNGuild. Our results indicated that *Ascomycota* and *Basidiomycota* were the most abundant fungal phyla across all STPs. The predominant fungal genera in STPs were Pichia, Sterigmatomyces, and Mortierella. The α-diversity varied significantly across the STPs based on observed, Fisher, and Shannon indices. Using SparCC cooccurrence network analysis, significant positive correlations of 58.5% and negative connections of 41.5% were obtained among fungal genera identified in STPs. Furthermore, the functional predictions by FUNGuild determined that STPs possessed high abundances of saprotroph and pathotroph-saprotroph-symbiotroph fungal trophic groups. At the functional guild level, the qiwam samples contained high abundances of soil saprotrophs, while plant pathogens were prevalent in pan-masala samples. These results suggest that various fungal populations reside in STPs and interrelate with each other and can contribute to the synthesis of TSNAs. This study has established the basis for future large-scale investigations of STP-associated mycobiota and the impact of such mycobiota in oral carcinogenesis in STP users via inflammation and carcinogens (TSNAs and mycotoxins).

**IMPORTANCE** Smokeless tobacco products (STPs) contain complex microbial communities that influence the synthesis of carcinogens, such as tobacco-specific nitrosamines (TSNAs). Research on STP-associated bacterial populations revealed connections between bacterial metabolism and TSNA synthesis. The abundance of the fungal population may also have an impact on the production of TSNAs. This study examined STPs popularly used in India, and diverse fungal communities were identified in these STPs. Pichia, Sterigmatomyces, and Mortierella were the predominant fungal genera in the STPs. High abundances of saprotroph and pathotroph-saprotroph-symbiotroph trophic groups in STPs could affect the degradation of tobacco products and the synthesis of TSNAs.

## INTRODUCTION

Globally, there are >300 million smokeless tobacco (SLT) users. The majority are in Southeast-Asian countries (>85%), but the use of SLTs is also prevalent in European and African countries and the United States (1–4). In India, there were 199.4 million SLT users reported in a global survey, and among them, 13.7 million users were below 21 years of age (5). Several chronic diseases, such as cancer, hypertension, heart diseases, stroke, and peptic ulcer, are associated with SLT use due to the presence of harmful substances, including tobacco-specific nitrosamines (TSNAs), polycyclic aromatic hydrocarbons [e.g., benzo(*a*)pyrene], and heavy metals (6–9). A large number of premature deaths due to SLT consumption were reported, contributing to increased disease burdens in the low- and low-middle-income groups of countries (10). TSNAs are the most abundant carcinogens in smokeless tobacco products (STPs) and can transform normal cells into cancerous cells (11). TSNA levels were found to be higher in STPs available in South-Asian countries than in those of Western countries (12). The levels of TSNAs in STPs depend upon tobacco constituents, moisture, temperature, pH, environmental conditions, storage conditions, curing processes, and aging of the products (12–19). Furthermore, the abolition of STP-associated microbiota by heating or other methods leads to low TSNA levels, suggesting a role of microbes in TSNA synthesis (17, 20, 21).

Worldwide, diverse forms of STPs are used, including moist/dry snuff, snus, chimó, nass (naswar), toombak, shammah, iq’mik, rapé, afzal, pituri, khaini, gutkha, qiwam (khiwam), zarda, gul, and loose powdered tobacco leaves (1). In India, the most abundantly consumed STPs are khaini, moist snuff, snus, pan-masala, zarda, gul, and qiwam (22). These STPs are dissimilar in their compositions and possess high levels of TSNAs (23, 24). Complex communities of bacterial species were identified in various categories of STPs (25–29). However, fungal communities are not well characterized in STPs, although these fungi have significant roles in the synthesis of carcinogens and as sources of mycotoxins (18, 30). The smoked products, such as cigarettes, showed increased growth of fungi as determined by high levels of ergosterol (a biomarker for fungi) when placed in highly humid conditions (31). A culture method showed the presence of several opportunistic and antifungal-resistant fungi, including Aspergillus, Penicillium, Mucor, Sepedonium, and Trichophyton, in Pakistani STPs that were related to khiwam (qiwam), gutkha, mawa, naswar, patti, and mainpuri (32). Aspergillus fumigatus was detected by a culture method in a gutkha product available in southern India (33). Furthermore, a metagenomic study on age-cured tobacco leaves showed the presence of fungal species belonging to the orders Incertae_sedis_*Eurotiomycetes*, *Wallemiales*, *Sporidiobolales*, *Capnodiales*, and *Eurotiales*, an unclassified *Ascomycota*, and an unidentified *Eurotiomycetes* in ascending order of relative abundance (16). The whole-metagenome approach identified fungal species belonging to the genera Alternaria and Aspergillus in American moist and dry STPs (34).

Based on the findings described above, contamination of Indian STPs with fungi needs to be investigated. Therefore, the fungal community compositions of such STPs were investigated by high-throughput sequencing, and a detailed and comprehensive analysis was performed by using the web-based tool MicrobiomeAnalyst (35). The ecological functions of STP-associated mycobiomes were determined by using FUNGuild (36).

## RESULTS

### Fungal community composition and diversity in STPs.

After processing of raw sequencing data (adaptor trimming and filtering out low-quality reads), 2,064,434 total read counts of fungal sequences were obtained, with an average of 98,306 per sample. A total of 1,275 operational taxonomic units (OTUs) were identified in all smokeless tobacco products (STPs). The Good’s coverage index was calculated for each STP and found to be >99% for all samples, confirming that the majority of true fungal biodiversity was covered by sequencing results in each STP. Furthermore, rarefaction curves showed increasing fungal species richness as the sequencing intensity increased and confirmed the accuracy of the view of the fungal diversity. The saturation of the rarefaction curve suggests that the maximum number of fungal species were identified in STPs. The highest levels of species richness were observed in samples of qiwam (Q2 and Q3), pan-masala (PM3), and gul (G1 and G2) products.

**FIG 1:**
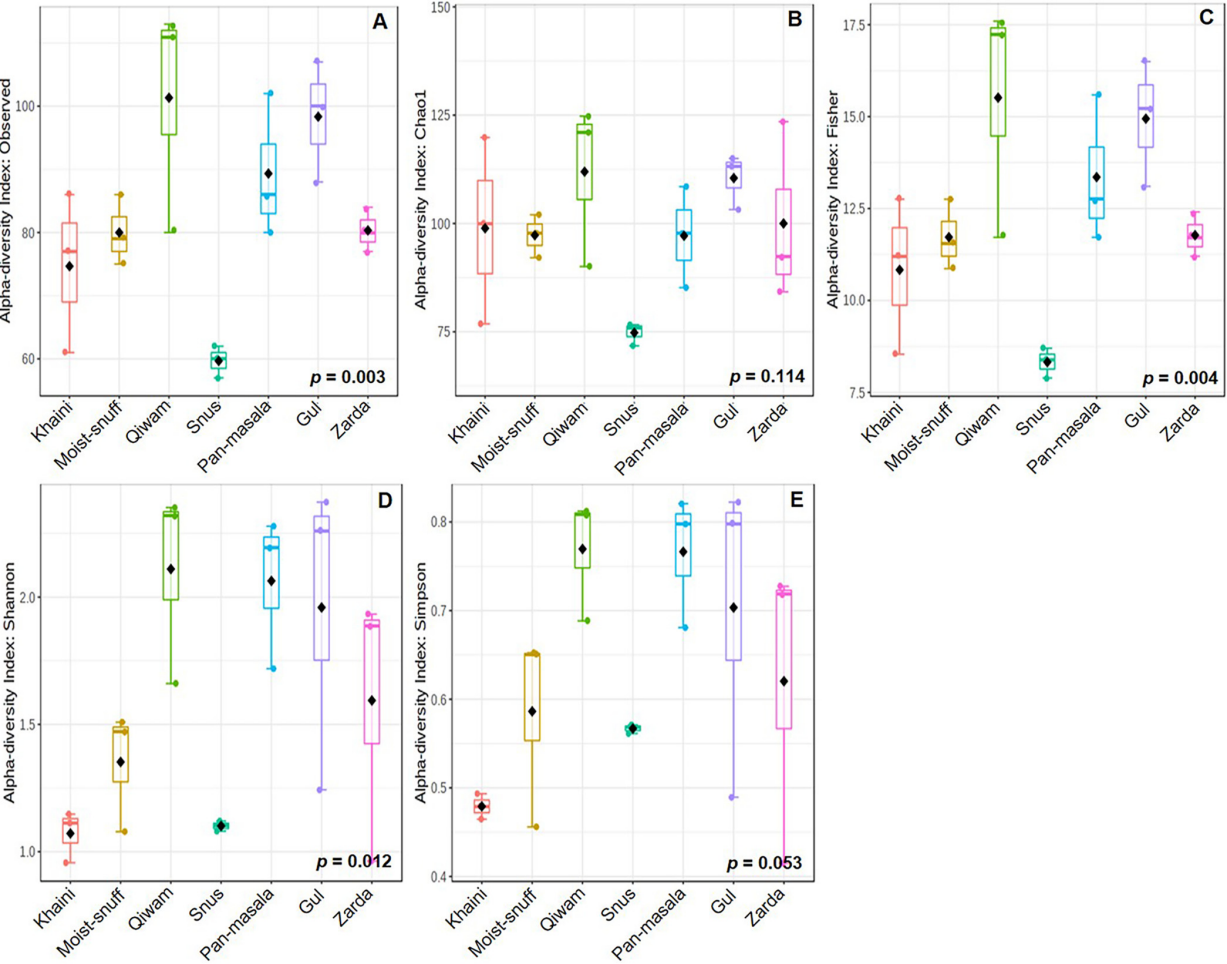
The α-diversities of fungal communities present in smokeless tobacco products. The box plots show the α-diversities of the fungal communities in the seven different types of smokeless tobacco products, namely, khaini, moist snuff, qiwam, snus, pan-masala, gul, and zarda, employing observed (A), Chao1 (B), Fisher (C), Shannon (D), and Simpson (E) indices. The boxes in each graph represent the ranges within 1.5 interquartile range (IQR), with median values shown by the lines in the boxes and mean values by black diamonds. Each colored dot represents the value for an individual sample. ANOVA was applied to determine the differences between the mean values of all groups.

Within-sample diversity (α-diversity) is defined in terms of richness (total number of fungal species) and evenness (relative abundances of fungal species within a sample). Significant differences in the fungal α-diversity were seen in the observed (total number of assigned OTUs to taxa per sample) (*P* = 0.003), Fisher (*P* = 0.004), and Shannon (*P* = 0.012) indices but not in the Chao1 (*P* = 0.114) and Simpson (*P* = 0.053) indices, obtained using the web-based tool MicrobiomeAnalyst (Fig. 1). The values for the Fisher index were highest for qiwam (mean ± standard error of the mean [SEM], 15.51 ± 1.902) and lowest for snus (mean ± SEM, 8.323 ± 0.235) samples (Fig. 1C). The qiwam samples had a significantly higher Shannon index value (mean ± SEM, 2.111 ± 0.225), whereas khaini samples had the lowest Shannon index (mean ± SEM, 1.072 ± 0.058) (Fig. 1D). Similarly, qiwam (mean ± SEM, 0.7696 ± 0.040) and khaini (mean ± SEM, 0.4791 ± 0.008) samples had the maximum and minimum Simpson values, respectively (Fig. 1E).

**FIG 2:**
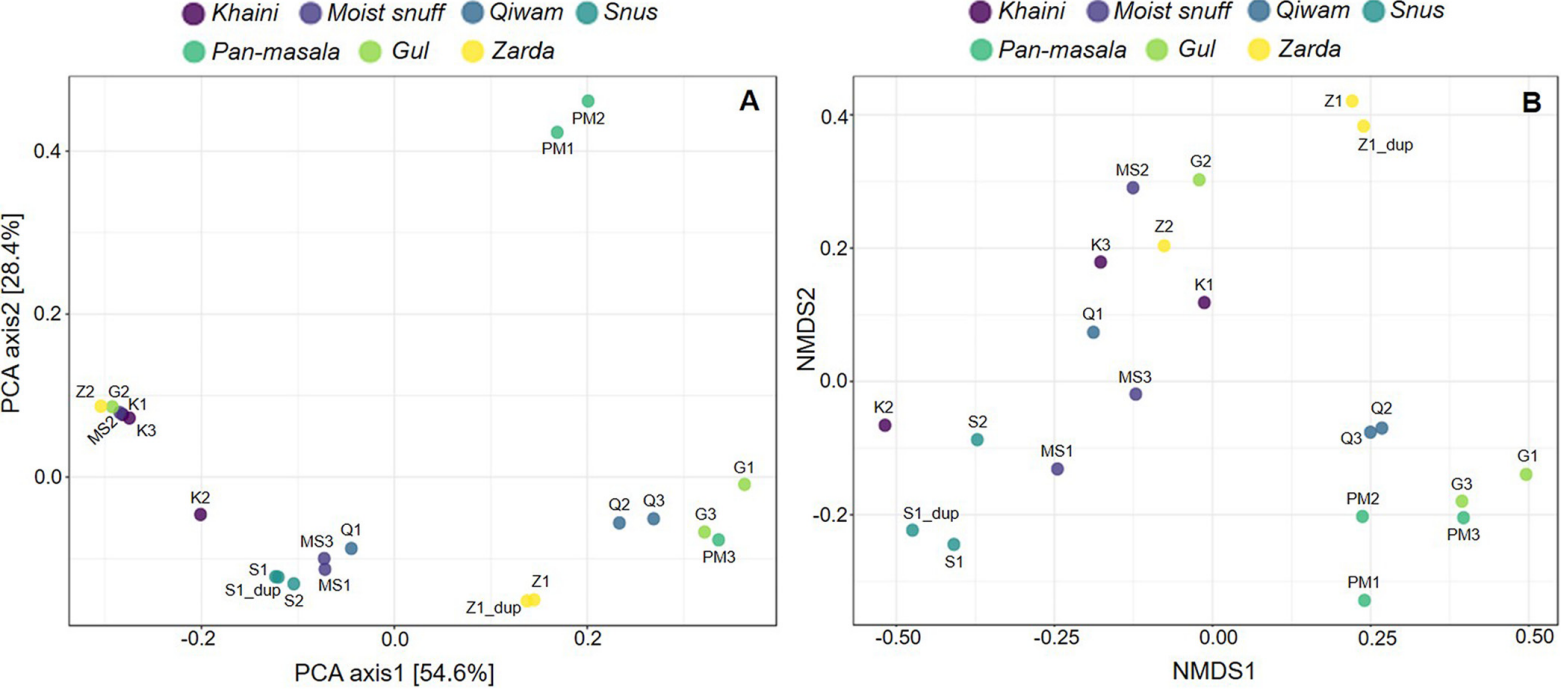
The β-diversities of fungal communities identified in smokeless tobacco products. Principal-component analysis (PCA) and nonmetric multidimensional scaling (NMDS) of the fungal communities derived from the Bray-Curtis index. (A) PCA plot showing the distances of fungal communities among khaini, moist snuff, qiwam, snus, pan-masala, gul and zarda. PERMANOVA, *F* = 2.602; *R*^2^ = 0.52722; *P < *0.012. (B) NMDS plot showing the distances of fungal communities among khaini, moist snuff, qiwam, snus, pan-masala, gul, and zarda samples. PERMANOVA, *F* = 2.602, *R*^2^ = 0.52722; *P < *0.012; [NMDS stress] = 0.14724. The product groups are identified by different colors as indicated above the plots. Each dot represents one sample.

The β-diversity analysis distributed the STPs into distinct clusters (Fig. 2). Principal-component analysis (PCA) of fungal communities displayed a 54.6% variation on axis 1 and 28.4% on axis 2 for all STPs (Fig. 2A). The compositions of fungal diversity of khaini samples K1 and K3, moist snuff sample MS2, gul sample G2, and zarda sample Z2 were found to be similar, and the same also occurred for moist snuff samples MS1 and MS3 and qiwam sample Q1 (Fig. 2A). The Q2 and Q3 samples showed a close association with each other and therefore had more-related mycobiomes. Pan-masala samples PM1 and PM2 exhibited a similar relationship, whereas PM3 showed a resemblance to sample G3. Next, snus samples S1, S1_dup, and S2 were clustered together, whereas one khaini sample (K2) and one gul sample (G1) did not cluster with other samples (Fig. 2A). Nonmetric multidimensional scaling (NMDS) also confirmed the close association between K1, K3, MS2, G2, and Z2, while other samples of qiwam, moist snuff, snus, pan-masala, gul, and zarda clustered separately (Fig. 2B). The statistical method permutational multivariate analysis of variance (PERMANOVA) revealed that the fungal communities across the STPs were significantly divergent (PERMANOVA, *F* = 2.602; *R*^2^ = 0.52722; *P < *0.012). Another nonparametric multivariate statistical test, analysis of group similarities (ANOSIM), showed that the fungal compositions present in all STPs were significantly dissimilar (ANOSIM test, *R* = 0.32678; *P < *0.004). In contrast, the homogeneity of group dispersions (PERMDISP) test of STPs was nonsignificant (*F* = 0.70549; *P *= 0.650), which suggested that differences in composition were not due to differences in multivariate dispersion.

### Taxonomic distribution of fungi identified in STPs.

**FIG 3:**
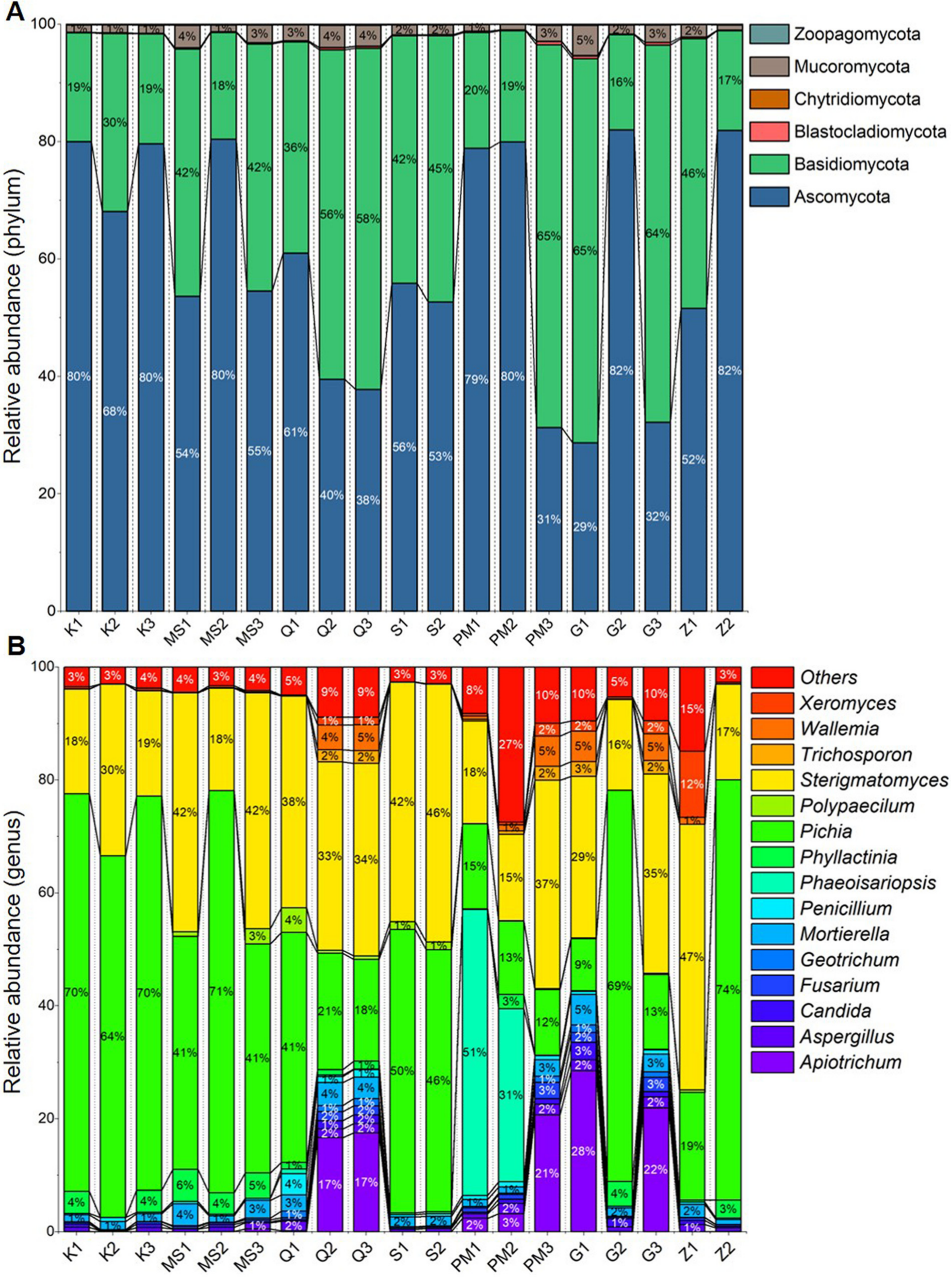
Fungal taxonomy identified in smokeless tobacco products. (A) The stacked bars show the relative abundances of fungal phyla identified in each smokeless tobacco product. Phyla represented by more than 10 OTUs are shown. Each fungal phylum is symbolized by a different color in the stacked bar graphs, with the boundary lines connecting the bars. (B) The stacked bars show the relative abundances of fungal genera identified in each smokeless tobacco product. Genera with high abundances are shown. Each fungal genus is symbolized by a different color in the stacked bar graphs, with the boundary lines connecting the bars. The entire set of relative abundances for each product was calculated as 100%.

The total OTUs were clustered and classified into 7 phyla, 30 classes, 78 orders, 190 families, and 451 genera. *Ascomycota* and *Basidiomycota* were the major dominant fungal phyla in all STPs (Fig. 3A). The relative prevalences of *Ascomycota* ranged from 68 to 80% in khaini, 54 to 80% in moist snuff, 38 to 61% in qiwam, 53 to 56% in snus, 31 to 80% in pan-masala, 29 to 82% in gul, and 52 to 82% in zarda samples. *Basidiomycota* relative abundances ranged from 19 to 30% in khaini, 18 to 42% in moist snuff, 36 to 58% in qiwam, 42 to 45% in snus, 19 to 65% in pan-masala, 16 to 65% in gul, and 17 to 46% in zarda samples (Fig. 3A). The rates of occurrence of *Mucoromycota* phyla ranged from 1 to 4% in the majority of STPs. However, the proportions of other fungal phyla, including *Blastocladiomycota*, *Chytridiomycota*, and *Zoopagomycota*, were less than 1% (Fig. 3A).

The maximum dominance at the class level was observed for *Saccharomycetes* (13 to 75%), followed by *Agaricostilbomycetes* (14 to 45%). The other noticeable classes observed were *Dothideomycetes*, *Eurotiomycetes*, *Leotiomycetes*, *Sordariomycetes*, and *Tremellomycetes* in a few STPs. The classes were again categorized into 33 orders, with the most prevalent being *Saccharomycetales* (13 to 75%) and *Agaricostibales* (14 to 45%). Furthermore, 68 families were noticed in STPs, of which the highly abundant families were *Pichiaceae* (11 to 75%), *Agaricostilbaceae* (14 to 46%), and *Trichosporanaceae* (33% in G1, 27% in G3, 26% in PM3, 22% in Q3, 21% in Q1, and 19% in PM2). The abundances of *Mycosphaerellaceae* were found to be high in two samples of pan-masala (46% in PM1 and 28% in PM2).

At the genus level, the most abundant genus was Pichia and the relative prevalences of Pichia were significantly high in samples of most STPs, including Z2 (74%), MS2 (71%), K1 (70%), K3 (70%), G2 (69%), K2 (64%), S1 (50%), S2 (46%), MS1 (41%), and Q1 and MS3 (41%) (Fig. 3B). In contrast, samples such as G1 (9%), PM3 (12%), PM2 (13%), PM1 (15%), and Q3 (18%) exhibited low abundances of the Pichia genus. Sterigmatomyces was another abundant genus in STPs, and its relative prevalences were found to be high in Z1 (47%), S2 (46%), MS1, MS3, and S1 (42%), Q1 (38%), PM3 (37%), G3 (35%), Q3 (34%), and Q2 (33%) (Fig. 3B). The genus Apiotrichum was detected at noticeable levels in G1 (28%), G3 (22%), PM3 (21%), and Q3 and Q2 (17%) (Fig. 3B).

**FIG 4:**
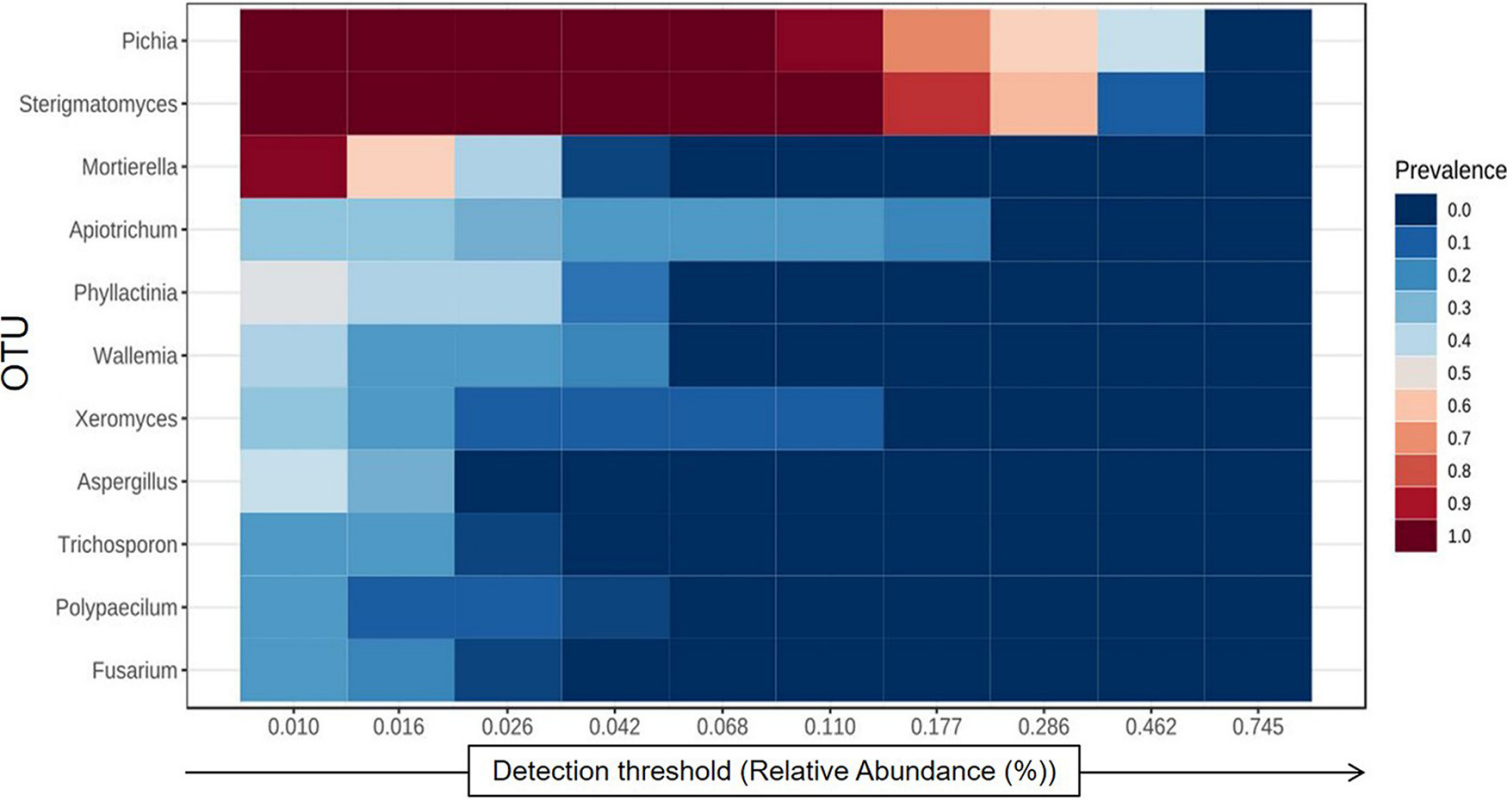
Core mycobiome of smokeless tobacco products. The core fungal genera in smokeless tobacco products were identified using as parameters sample prevalence (≥20%) and relative abundance (≥0.01%). The heatmap illustrates the detection thresholds and relative abundances of the most dominant fungal genera in the tested smokeless tobacco products. The color key shows the range of threshold relative abundances of the individual values.

Core microbiomes were measured as the microbial taxa shared among two or more samples from a particular host or environment (37). Despite interproduct variability, there was a core mycobiome identified in the STPs that remain unchanged in its composition across different sample groups based on sample prevalence and relative abundance (Fig. 4). The overall core fungal mycobiome of all STPs consisted of 11 genera: Pichia, Sterigmatomyces, Mortierella, Apiotrichum, Phyllactinia, Wallemia, Xeromyces, Aspergillus, Trichosporon, Polypaecilum, and Fusarium (Fig. 4). The core fungal communities of STPs were dominated by Pichia, Sterigmatomyces, and Mortierella (Fig. 4).

### Cluster analysis of fungal genera identified in STPs.

**FIG 5:**
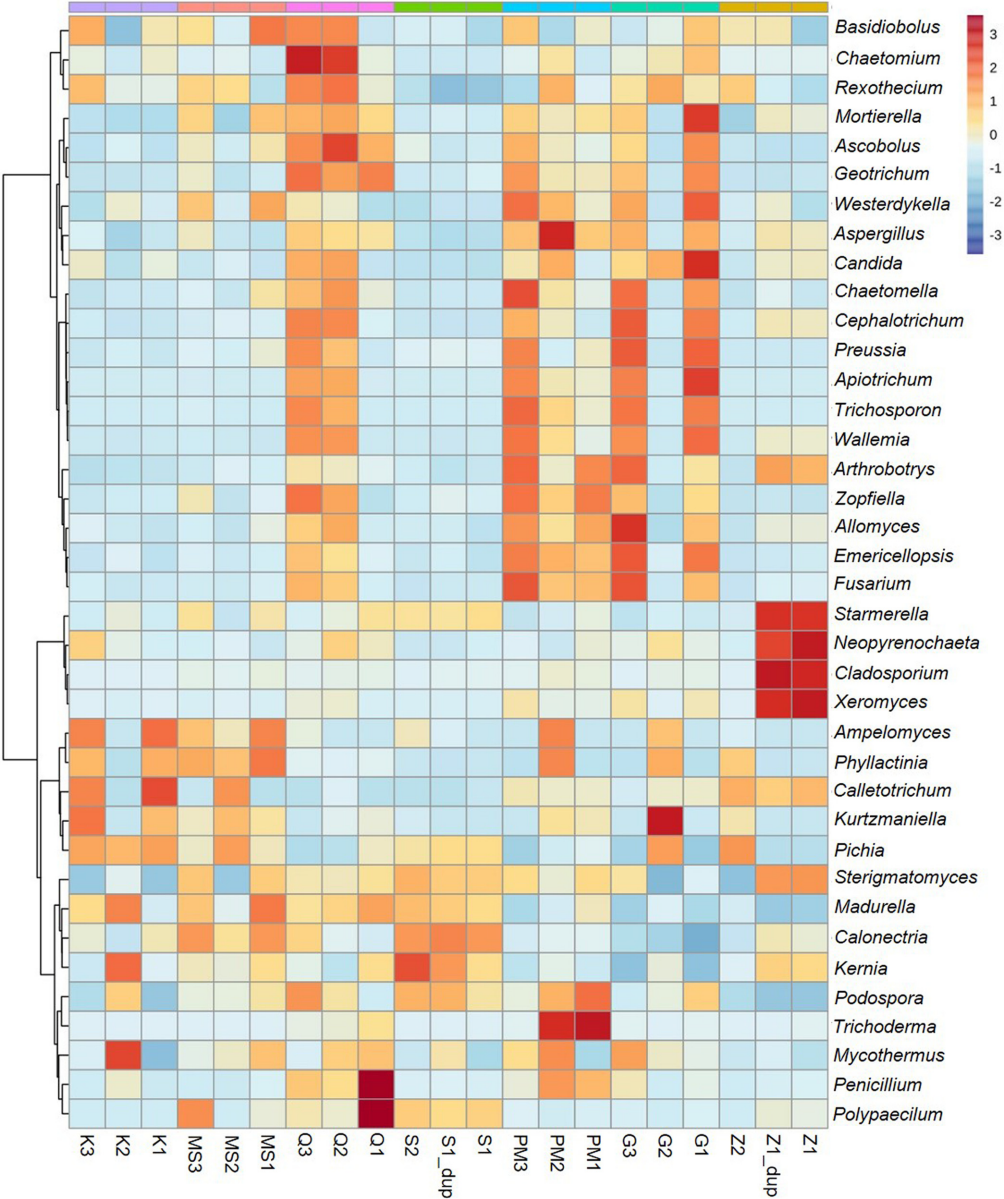
Cluster analysis of smokeless tobacco product-associated fungi. Heatmap shows fungal genera with their prevalences in all smokeless tobacco product groups: (i) khaini, (ii) moist snuff, (iii) qiwam, (iv) snus, (v) pan-masala, (vi) gul, and (vii) zarda. Ward’s clustering algorithm along with Euclidean distance was used to generate the hierarchical tree. Each column represents a sample, and each row the indicated fungal genus with relative abundances indicated by color as shown in the key.

The abundance pattern of fungal OTUs coupled with cluster analysis is represented in the form of a heatmap (Fig. 5). The fungal genera enriched in sample K1 were Colletotrichum and Ampelomyces, while Mycothermus, Kernia, and Madurella were enriched in K2 and Kurtzmaniella, Colletotrichum, and Ampelomyces were enhanced in K3 (Fig. 5). Certain fungal genera were enriched in moist snuff samples, such as Basidiobolus, Phyllactinia, Madurella, and Ampelomyces in MS1 and Polypaecilum, Calonectria, and Phyllactinia in MS3. The qiwam category demonstrated the dominance of Penicillium, Polypaecilum, and Geotrichum genera in Q1, Ascobolus, Chaetomium, Basidiobolus, and Rhexothecium genera in Q2, and Chaetomium, Geotrichum, Zopfiella, and Basidiobolus genera in Q3 (Fig. 5). The fungal genera Trichoderma, Podospora, Zopfiella, and Arthrobotrys were found to be enriched in PM1, Aspergillus, Trichoderma, Phyllactinia, and Ampelomyces in PM2, and Chaetomella, Fusarium, Trichosporon, and Arthrobotrys in PM3. For gul samples, Mortierella, Candida, Apiotrichum, and Westerdykella were abundant in G1, whereas Kurtzmaniella was prominent in G2 (Fig. 5). Sample G3 showed enrichment of Allomyces, Cephalotrichum, Preussia, Fusarium, and Emericellopsis fungal genera, whereas Z1 showed prevalences of Neopyrenochaeta, Xeromyces, Starmerella, and Cladosporium (Fig. 5).

### Biomarker examination of STPs.

**FIG 6:**
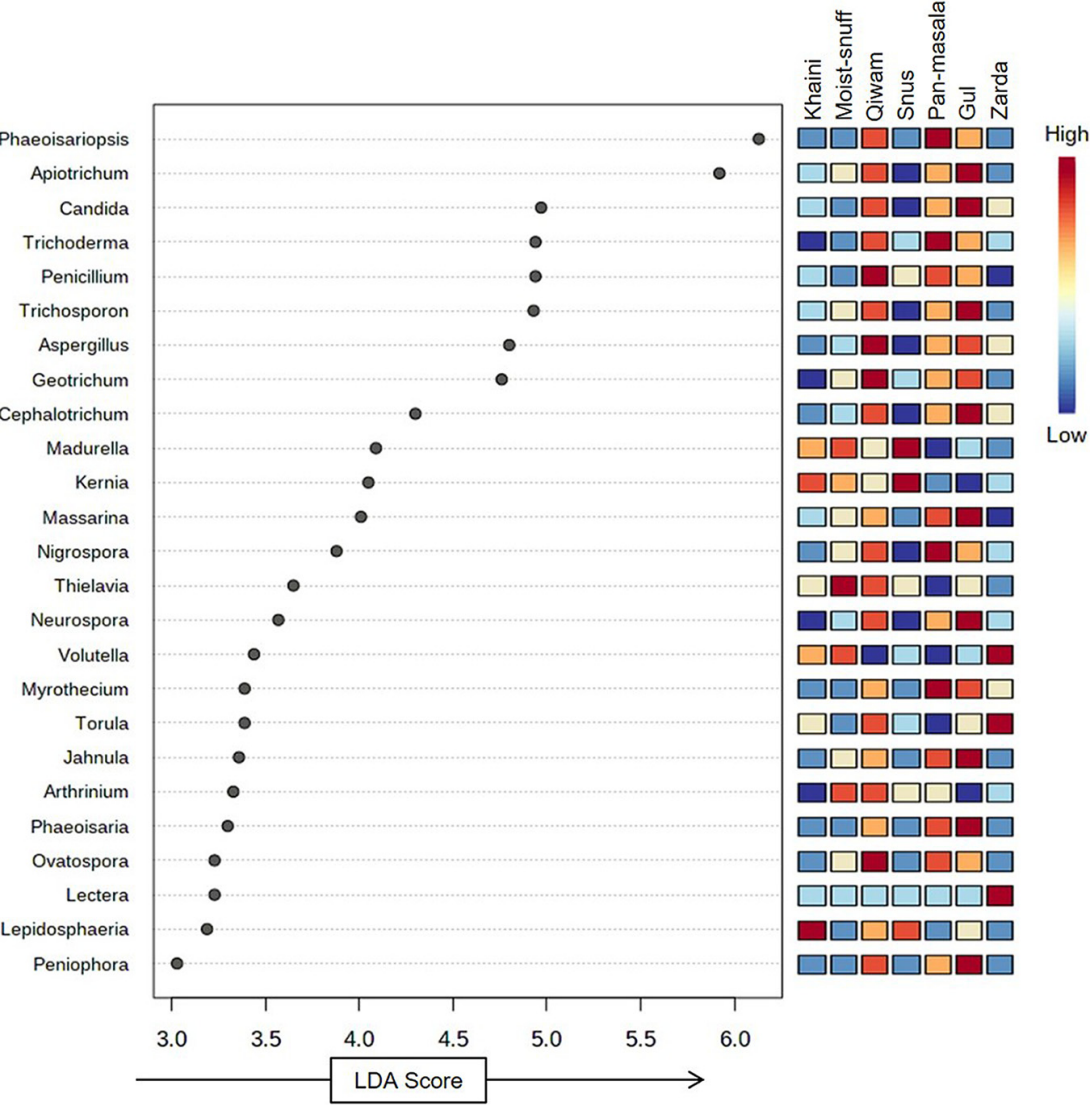
Biomarker analysis of smokeless tobacco product-linked mycobiomes. Linear discriminant analysis effect size (LEfSe) analysis of mycobiomes present in smokeless tobacco product groups (i) khaini, (ii) moist snuff, (iii) qiwam, (iv) snus, (v) pan-masala, (vi) gul, and (vii) zarda. The 25 most significant genera were ranked in declining order according to their LDA scores (*x* axis). LEfSe parameters: *P* value cut off = 0.05 and log LDA score = 2.0.

The LEfSe (linear discriminant analysis effect size) analysis found a total of 25 significant OTUs, and Lepidosphaeria was the taxon that contributed the most OTUs for khaini samples, whereas Thielavia contributed the most for the moist snuff samples (Fig. 6). Furthermore, Penicillium, Aspergillus, Geotrichum, and Ovatospora were found to be the genera contributing the most OTUs for qiwam samples (Fig. 6). Moreover, the linear discriminant analysis (LDA) scores of Madurella and Kernia were highest for snus samples. Four genera of fungi, namely, Phaeoisariopsis, Trichoderma, Nigrospora, and Myrothecium, were significantly enriched in pan-masala samples; nine genera, Apiotrichum, Candida, Trichoderma, Neurospora, Cephalotrichum, Massarina, Jahnula, Phaeoisaria, and Peniophora, were significantly enriched in gul samples; and three fungal genera, Volutella, Torula, and Lectera, were significantly enriched in zarda samples (Fig. 6).

### SparCC correlation network.

**FIG 7:**
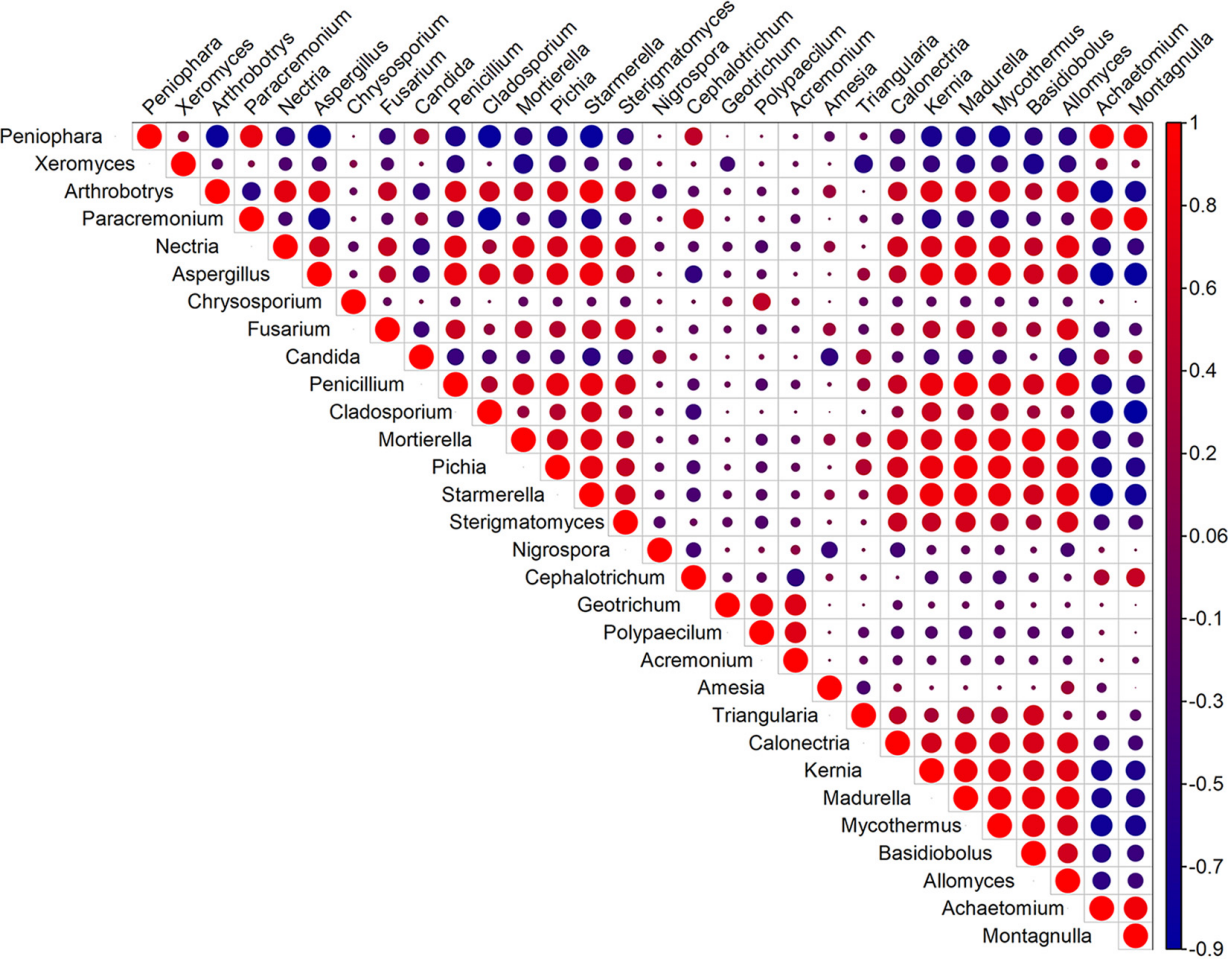
Cooccurrence of fungal genera in smokeless tobacco products. The SparCC correlations of selected fungal genera were generated and plotted in a heatmap. The scale bar on the right of the plot shows the calculated positive and negative correlation values used to generate the heatmap. The correlation threshold was >0.3, and the *P* value was set at <0.05.

SparCC was used to calculate the correlations between OTU prevalences in the mycobiome data (38). In total, 1,398 positive (58.5%) and 992 negative (41.5%) significant correlations (|correlation coefficient [=corr]| > 0.3 and *P* ≤ 0.05) were found among fungal genera. The highest positive correlations were found between Candida and Triangularia (corr = 1, *P* = 0.009). The genus Aspergillus was found to be correlated positively with Starmerella (corr = 0.85, *P* = 0.029), Calonectria (corr = 0.8211, *P* = 0.049), Penicillium (corr = 0.7821, *P* = 0.009), and Mortierella (corr = 0.7193, *P* = 0.009), while it was negatively correlated with Cystobasidium (corr = −0.8866, *P* = 0.009) and Jahnula (corr = −0.7852, *P* = 0.019) (Fig. 7). The Candida genus was also positively correlated with Scopulariopsis (corr = 0.9534, *P* = 0.009) and negatively correlated with Amesia (corr = −0.9475, *P* = 0.009), Mortierella (corr = −0.8022, *P* = 0.029), Fusarium (corr = −0.7443, *P* = 0.049), Cladosporium (corr = −0.6528, *P* = 0.039), Starmerella (corr = −0.6614, *P* = 0.009), and Tortispora (corr = −0.6778, *P* = 0.049) (Fig. 7). The fungal genus Mortierella showed positive association with Starmerella (corr = 0.9898, *P* = 0.039), Allomyces (corr = 0.9463, *P* = 0.039), and Stachybotrys (corr = 0.9199, *P* = 0.029), while it correlated negatively with Xeromyces (corr = −0.8149, *P* = 0.009) (Fig. 7). Another important genus, Fusarium, displayed positive concurrence with Allomyces (corr = 0.9453, *P* = 0.029), Starmerella (corr = 0.9238, *P* = 0.049), Sterigmatomyces (corr = 0.8982, *P* = 0.0297), Mortierella (corr = 0.854, *P* = 0.049), and Stachybotrys (corr = 0.8398, *P* = 0.049) (Fig. 7). The genus Pichia was positively correlated with Starmerella (corr = 0.8667, *P* = 0.039) and negatively correlated with Apiotrichum (corr = −0.8888, *P* = 0.009) and Cephalotrichum (corr = −0.6379, *P* = 0.029) (Fig. 7).

### Functional potentials of the fungal population associated with STPs.

**FIG 8:**
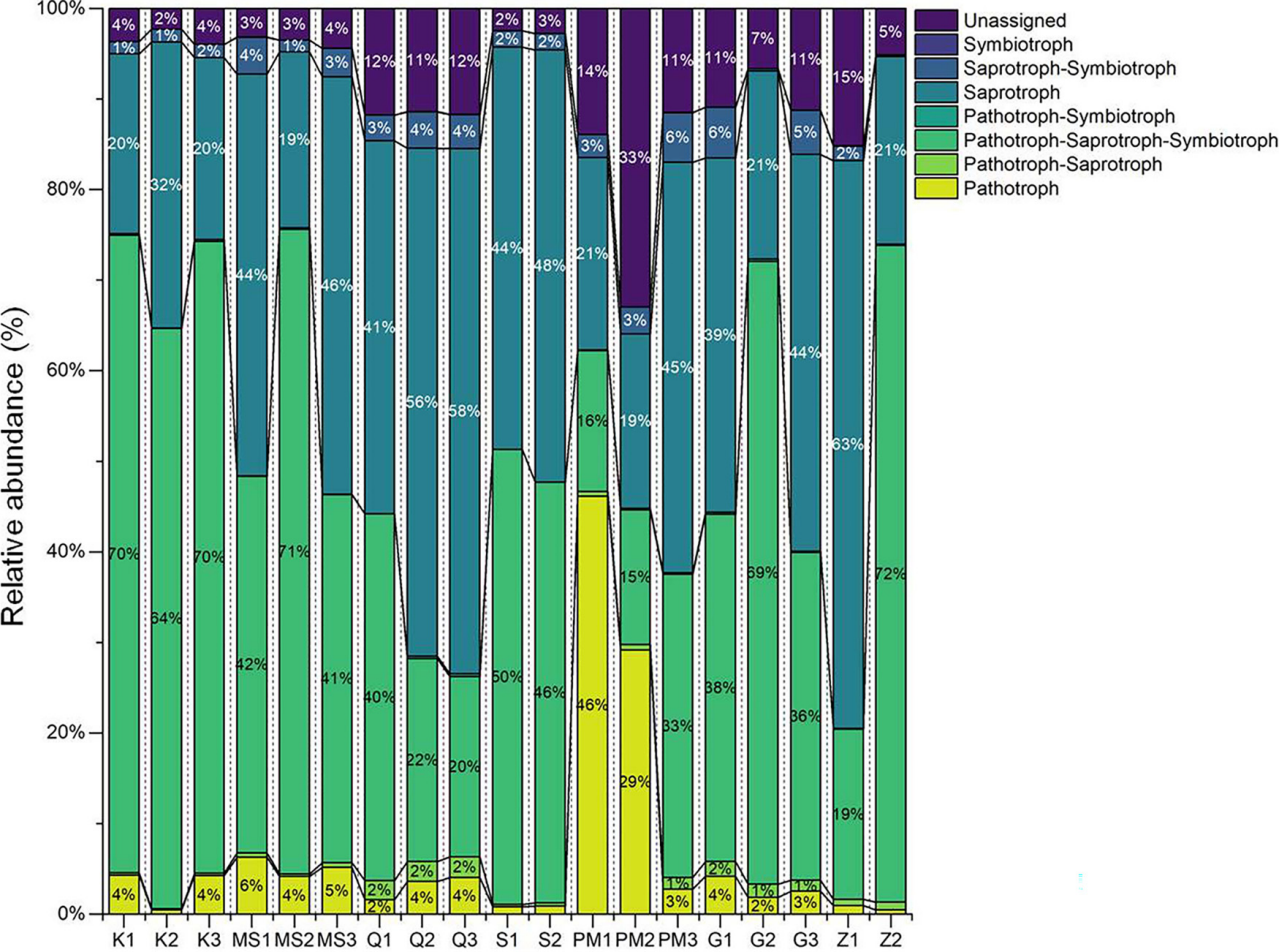
Relative abundances of fungal trophic modes in smokeless tobacco products. Each fungal trophic mode is symbolized by a different color in the stacked bar graphs, with the boundary lines connecting the bars. The entire set of relative abundances for each product was calculated as 100%.

**FIG 9:**
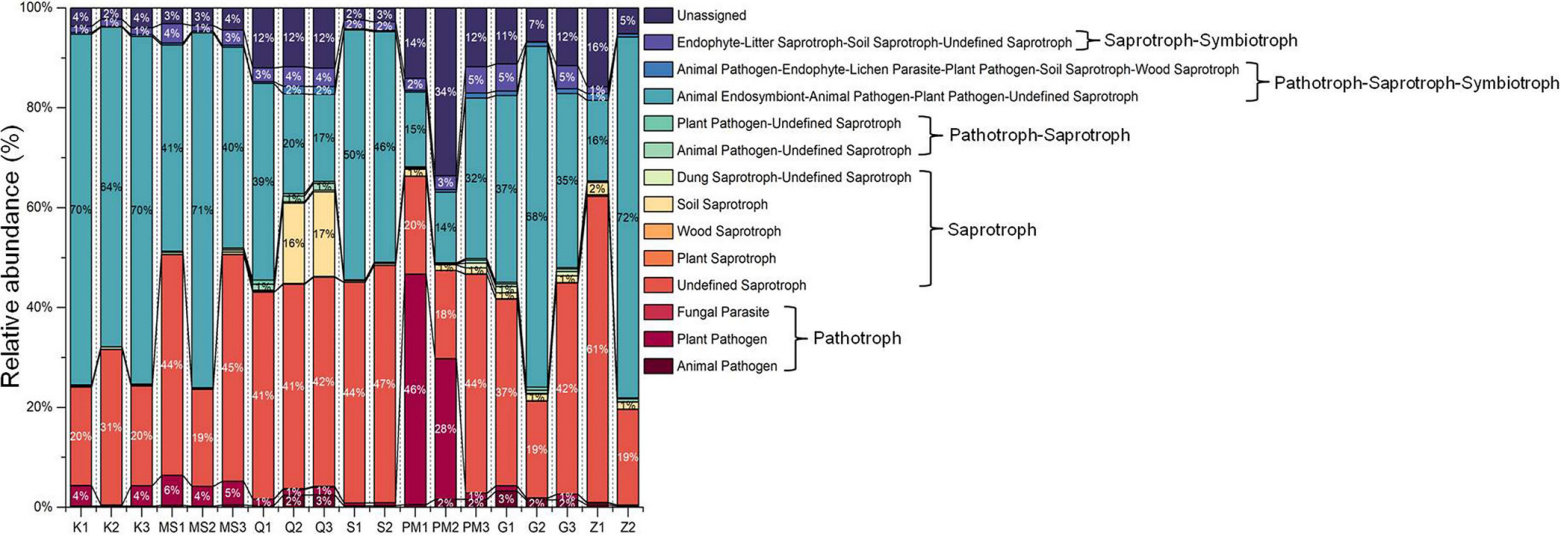
Relative abundances of fungal functional groups (guilds) in smokeless tobacco products. Each fungal guild is symbolized by a different color in the stacked bar graphs, with the boundary lines connecting the bars. The entire set of relative abundances for each product was calculated as 100%.

The associations between the fungal communities of STPs and their functional groups (guilds) were inferred by using the FUNGuild database (36). A total of 1,275 OTUs that were identified in STPs were divided into 910 matched OTUs (information originated from primary research) and 365 unmatched OTUs. The main ecological fungal trophic modes, i.e., pathotrophs, saprotrophs, and symbiotrophs, were identified in all STPs (Fig. 8). The relative abundances of pathotrophs-saprotrophs-symbiotrophs were higher than those of other trophic modes in the majority of STP samples (72% in Z2, 71% in MS2, 70% in K1 and K3, 69% in G2, 64% in K2, and 50% in S1) (Fig. 8). The relative occurrences of saprotrophs were found to be elevated in Z1 (63%), Q3 (58%), Q2 (56%), S2 (48%), MS3 (46%), PM3 (45%), MS1 and G3 (44%), Q1 (41%), and G1 (39%). The dominance of pathotrophs was found to be considerably elevated in pan-masala samples (33% in PM2 and 14% in PM1) compared to their levels in other products (Fig. 8). Furthermore, the prevalences of unassigned groups were notably high in pan-masala, qiwam, gul, and zarda samples compared to their levels in khaini, moist snuff, and snus samples (Fig. 8). Among the 71 functional guilds identified, the undefined saprotroph and animal endosymbiont-animal pathogen-plant pathogen-undefined saprotroph guilds were highly abundant in most of the STPs, except for pan-masala samples PM1 and PM2, which exhibited high proportions of plant pathogens (Fig. 9). Furthermore, qiwam samples possessed significantly increased abundances of soil saprotrophs compared to the levels in other STPs (Fig. 9).

### Mycotoxins in STPs.

The International Agency for Research on Cancer (IARC) has classified naturally occurring aflatoxins as carcinogenic to humans (group 1) and ochratoxin A (OTA) as a possible human carcinogen (group 2B) (39). Aflatoxins are immunosuppressive, hepatotoxic, and carcinogenic molecules and are mainly synthesized by fungi belonging to the genus Aspergillus (40). OTA is produced by members of the genera Aspergillus and Penicillium and has been found to induce health effects via nephrotoxicity, hepatotoxicity, immunotoxicity, neurotoxicity, and teratogenicity (40). Using the liquid chromatography-tandem mass spectrometry (LC-MS/MS) method for detection of selected mycotoxins in STP samples (*n* = 10) showed that the levels of aflatoxin B1, B2, G1, and G2 and OTA were below the limit of detection (LOD; <0.5 ppb) in all samples tested.

## DISCUSSION

It has been demonstrated that complex microbial communities are present in STPs and that these microorganisms can play an essential role in the synthesis of TSNAs through their metabolic activities (18, 27). The presence of STP-associated fungi was previously established by culture-dependent methods (32, 33, 41, 42). However, very limited research focusing on the identification and determination of the functional potential of the fungal populations has been performed in STPs (34). Therefore, we executed the identification of members of the mycobiomes and a comprehensive comparison of fungal communities in STPs belonging to seven major categories: khaini, moist snuff, qiwam, snus, pan-masala, gul, and zarda. These products are popularly consumed in India and are considered to be one of the major causes of the high occurrence of oral cancer in India (43).

The α-diversities of the fungal populations in STPs were found to be significantly different, and qiwam, pan-masala, and gul samples showed high α-diversity indices (observed, Fisher, and Shannon). The fungal β-diversities between different samples were analyzed by principal-component analysis (PCA) and NMDS, and it was observed that most of the samples of the seven categories of products, khaini, moist snuff, qiwam snus, pan-masala, gul, and zarda, had distinct fungal β-diversities. These differences could be due to different manufacturers of samples belonging to the same product category. The phyla *Ascomycota* and *Basidiomycota* were previously found to be the prevalent phyla on tobacco leaves (44). In this study, *Ascomycota* was the predominant phylum in STPs, followed by *Basidiomycota*, which corroborated a previous study on aged tobacco leaves collected from Baoshan, Yunnan Province, in China (45). However, a whole-metagenome analysis of American snuff (moist/dry) products showed that *Ascomycota* was the most common fungal phylum (34). Both *Ascomycota* and *Basidiomycota* are the most abundant and diverse fungal phyla involved in degradation activities (decomposers) (46–48). The high prevalences of *Ascomycota* and *Basidiomycota* in all STPs may be due to their selection during the curing and storage of tobacco leaves and contamination of these products during manufacturing. At the family level, the American dry snuff products showed a prevalence of the family Aspergillaceae (*Ascomycota*), whereas we observed an abundance of the families *Pichiaceae* (*Ascomycota*) and *Agaricostilbaceae* (*Basidiomycota*) in Indian STPs (34). In tobacco or tobacco products, several fungal genera were identified, including Aspergillus, Candida, Fusarium, Cladosporium, Alternaria, and Acremonium, which can reduce nitrate into nitrite, which reacts with different alkaloids to form TSNAs (17, 18, 49, 50). These fungal species (i.e., Aspergillus) can be sources of mycotoxins, as previously identified in stored leaves of chewing tobacco (51). In this study, we observed that the core fungal genera that remained unchanged in their composition across different STPs were Pichia, Sterigmatomyces, Mortierella, Apiotrichum, Phyllactinia, Wallemia, Xeromyces, Aspergillus, Trichosporon, Polypaecilum, and Fusarium. These fungal genera are thought to play an important role in the microenvironment (niche) of STPs. Previous studies showed that fungi like Fusarium, Geotrichum, and Aspergillus, as well as other genera, could not only reduce nitrates to nitrites but also decompose proteins and increase the concentrations of amines in moldy foods, thus providing favorable conditions for the synthesis of carcinogenic nitrosamines (52).

Pichia is the largest fungal genus, ubiquitously present in natural habitats, and Pichia spp. also ferment several foods and beverages and have even found to be pathogens (53–56). Furthermore, Pichia spp. can produce several killer toxins (e.g., PMKT and PMKT2) that are lethal to other sensitive yeasts and filamentous fungi (57). Pichia spent medium suppresses the growth of Aspergillus, Candida, and Fusarium via a secretory protein, probably a mycotoxin (58). Hence, the abundance of the Pichia genus in Indian STPs can inhibit the growth of other fungi in STPs and may also eliminate oral fungal species. The antagonistic effect of Pichia on the oral mycobiome resident in SLT users can be further investigated to decipher Pichia’s role in STP-associated oral cancer. Additionally, because the abundance of the Pichia genus in STPs may inhibit the growth of other fungi (e.g., Aspergillus and Penicillium), the levels of mycotoxins may be reduced in STPs. Such an assumption can be correlated with our observations, as the abundances of mycotoxin-producing fungi were found to be less and the levels of mycotoxins were negligible (less than the LOD of 0.5 ppb) in most of the tested STPs.

Another abundant genus in Indian STPs was Sterigmatomyces. Recently, a few species of Pichia and Sterigmatomyces were found to survive under extreme environmental conditions, such as high salt (halophiles) (59, 60). A salt-tolerant basidiomycetous fungal species, Sterigmatomyces halophilus, was also discovered in the gut microbiota of wood-feeding termites (61). S. halophilus contains catalyzing reductase enzymes, and such reductase activity at a broad range of pH values (3.0 to 10.0) was exploited in the biodegradation of environmental pollutants like azo dyes (62). Furthermore, S. halophilus showed adsorption of heavy metals like cadmium, copper, iron, manganese, lead, and zinc from growth media supplemented with these metallic salts (63). These trace metals have also been reported in STPs, and therefore, the presence of S. halophilus in STPs may have a positive impact (64). Hence, Sterigmatomyces fungi have the metabolic capability to degrade a variety of chemicals, and future work is needed to ascertain whether these fungi are associated with TSNA formation in STPs. Furthermore, there is evidence that Sterigmatomyces is able to upregulate the expression of several immune-related genes and, therefore, may have a possible role in inflammation and disease progression if it comes in contact with the buccal mucosa upon STP consumption. S. halophilus derived β-glucan was able to increase cytokine gene expression in Pacific red snapper (Lutjanus peru)-derived leucocytes upon bacterial challenge (65). Similar immune response generation was observed in the pathogen-infected fish gilthead seabream (Sparus aurata L.) fed with an S. halophilus-supplemented diet (66).

In our study, the third most abundant fungal genus was Mortierella. The numerous strains of the Mortierella genus are saprotrophic, found in normal as well as extreme, hostile environments, and they have a significant role as valuable decomposers in agricultural soil (67). Hence, the occurrence of Mortierella spp. in the tobacco product microenvironment is not surprising, as they can survive harsh conditions and utilize cellulose, hemicellulose, and chitin as carbon sources (67). Therefore, it can be hypothesized that members of the Mortierella genus can survive in tobacco products for a long duration and perform their metabolic activities. It will be an interesting line of research to evaluate the conversion of nitrate into nitrite (which reacts with tobacco alkaloids to form TSNAs) by the reductases [e.g., NAD(P)H:nitrate reductase, NaR] of extremophilic fungi, particularly Pichia, Sterigmatomyces, and Mortierella fungi, resident in Indian STPs.

Next, we determined the specific fungal taxa that had significantly higher relative abundances in each STP and could be used in future investigations on the determination of potential taxonomic markers in different STPs from different areas. According to the LEfSe results, nine genera, namely, Apiotrichum, Candida, Trichosporon, Neurospora, Cephalotrichum, Massarina, Jahnula, Phaeoisaria, and Peniophora, were abundant in gul samples, while the khaini and moist snuff samples each had only one fungal genus biomarker, Lepidosphaeria and Thielavia, respectively. The other contributing genera were Madurella and Kernia in snus samples, Penicillium, Aspergillus, Geotrichum, and Ovatospora for qiwam samples, Phaeoisariopsis, Trichoderma, Nigrospora, and Myrothecium for pan-masala samples, and Volutella, Torula, and Lectera for zarda samples. Notably, six of these genera (Candida, Aspergillus, Geotrichum, Penicillium, Trichosporon, and Fusarium) are opportunistic pathogenic fungi and previously reported to be associated with human diseases, including oral cancer, allergy, acute myelogenous leukemia, and fusariosis (68–72). Therefore, LEfSe analysis may be a useful tool for identifying key fungal taxa related to human pathogens present in STPs.

Furthermore, cooccurrence network analysis by SparCC was performed to unravel the potential relationships between the fungal taxa in the different STPs, and strong fungal abundance correlations were observed in the STPs. Significant positive correlations were observed among the fungi Aspergillus, Penicillium, Cladosporium, and Fusarium, which are well-known asthma allergens (73). Similar to this study, a correlation between Aspergillus and Cladosporium was observed in the airborne fungal spores in ambient particulate matter of North-East India, indicating that these fungi in Indian STPs can be enriched during air curing of the tobacco leaves (74). Nutrient availability was an important factor that primarily shaped the microbial community network. For example, “cherry-red” tobacco leaves, which have higher phytol content than ordinary tobacco leaves, had more decomposer fungi (75). This may explain the higher complexity of the fungal populations in STPs, as different products contain a variety of nutrient ingredients, such as areca nut, catechu, spices, glycerin, clove oil, camphor, etc. Furthermore, snus and other STPs were associated with high prevalences of asthma and respiratory symptoms (76, 77). Hence, fungi present in Indian STPs can cause asthma-like symptoms to develop in their users, which needs to be further investigated, as tobacco use is one of the leading risk factors for chronic obstructive pulmonary disease in India (78).

Furthermore, we exploited the FUNGuild database to predict functional guilds from the fungal OTUs identified in STPs. It was observed that most of the fungal OTUs in all STPs belonged to saprotroph and pathotroph-saprotroph-symbiotroph trophic groups, while the pathotroph trophic group was abundant in two samples of pan-masala. Furthermore, the majority of OTUs were assigned to either undefined saprotroph or animal endosymbiont-animal pathogen-plant pathogen-undefined saprotroph. The prevalence of undefined saprotroph guilds suggests that decomposer fungi were high in number across all STPs. This finding is consistent with results obtained previously where a FUNGuild analysis showed that saprophyte and pathotroph-saprotroph-symbiotroph were the dominant trophic modes in the fungal community during fermentation of cigar tobacco leaves (79). Another FUNGuild analysis of stored tobacco leaves revealed that the fungal community was comprised mainly of saprotrophs and pathotrophs, suggesting that the fungal community largely acquired nutrients by decomposing or damaging host cells (tobacco leaves) for their survival (79). Therefore, saprotrophic fungi can accelerate the decomposition of organic matter available in STPs and would aid the degradation of carbon and nitrogen compounds, resulting in the elevated levels of TSNAs (45, 80).

In this study, Pichia (animal endosymbiont-animal pathogen-plant pathogen-undefined saprotroph) was the most abundant genus in the STPs. A few studies have identified Pichia as an emerging opportunistic pathogen causing serious infections in immunodeficient patients and infants in neonatal intensive care units (53–55). An opportunistic pathogen, Pichia kudriavzevii (syn. Candida krusei syn. Issatchenkia orientalis), recognized as a fifth leading cause of yeast infections, was found to be resistant against fluconazole and showed increased resistance toward other antifungal drugs (81, 82). Furthermore, Pichia kudriavzevii had a significant association with plants, which can also be a possible reason for the presence of this genus in tobacco leaves and may play a role in oral pathogenesis (82). Furthermore, the plant pathogen guild was relatively abundant in pan-masala and noticeably observed in khaini, moist snuff, qiwam, and snus samples. This guild contains many fungi in the family *Erysiphaceae* that have been previously identified as abundant in healthy tobacco leaves (83).

Finally, in this study, we collected the STPs from a defined area due to travel restrictions during the coronavirus disease 2019 (COVID-19) pandemic. For a conclusive outcome, products (STPs) from other geographical areas of India need to be investigated, because the dynamics of microbial populations depend upon the climate, storage, and manufacturing conditions.

### Conclusion.

In summary, the diversity of fungal communities is an important component of STPs that is markedly different among different products. The genera Pichia, Sterigmatomyces, and Mortierella, belonging to phyla *Ascomycota*, *Basidiomycota*, and *Mucoromycota*, respectively, were the most abundant fungal genera in STPs. The core mycobiome has the potential to decompose the substrates present in STPs. Understanding the fungal communities associated with STPs will provide a basis to decipher the carcinogenic potential of STPs, as these fungi can metabolize the constituents of STPs and convert them into carcinogens, such as TSNAs. This work provides the first comprehensive description of the fungal community associated with Indian STPs and will provide a valuable benchmark for future studies aiming to identify the metabolic involvement of fungi in carcinogen synthesis and the induction of oral cancer in smokeless tobacco users.

## MATERIALS AND METHODS

### Selection of STPs, DNA isolation, and PCR amplification.

Different categories of smokeless tobacco products (STPs) (*n* = 19), including khaini (samples K1, K2, and K3), moist snuff (MS1, MS2, and MS3), qiwam (Q1, Q2, and Q3), snus (S1 and S2), pan-masala (PM1, PM2, and PM3), gul (G1, G2, and G3), and zarda (Z1 and Z2), were collected from the northern region of India. The STPs were kept at −20°C to restrain the further growth of fungal microorganisms.

To avoid environmental contamination, the outsides of the STP packages were UV treated and the products unpacked under a sterilized ambience. The genomic DNA of microbial communities was separated with a PowerSoil DNA isolation kit as instructed by the manufacturer (Qiagen, Bengaluru, India). Before PCR amplification, the quality of metagenomic DNA was determined by 1% agarose gel, and a NanoDrop instrument (Thermo Fisher, Bangalore, India) was used to check the purity and concentration of the metagenomic DNA. The internal transcribed spacer 1 (ITS1) segment of fungal ribosomal DNA was amplified using primers ITS1f/2043R (forward, 5′-TTGGTCATTTAGAGGAAGTAA-3′, and reverse, 5′-GCTGCGTTCTTCATCGATGC-3′) (84). The PCR mixture contained 40 ng metagenomic DNA, 10 pM each primer, high-fidelity DNA polymerase, 0.5 mM deoxynucleoside triphosphates (dNTPs), 3.2 mM MgCl_2_, and PCR enzyme buffer. The PCR amplification was performed with the following conditions: initial denaturation at 95°C for 180 s, followed by 25 cycles of 95°C for 15 s, 60°C for 15 s, and 72°C for 120 s and a final extension at 72°C for 10 min. The amplified ITS PCR product was purified and subjected to 2% agarose gel and NanoDrop analysis for a quality check. A PCR-amplified ultrapure water (microbial DNA-free water) sample was included as a negative control in the study. The PCR amplification of the negative-control sample did not produce detectable amplicons.

### Library preparation and targeted amplicon sequencing.

Initially, the unused primers were removed by purification of amplicons with AMPure beads (Beckman Coulter, Inc., Indianapolis, IN). Another PCR of 8 cycles was performed with Illumina bar-coded adapters to formulate the sequencing libraries. After that, prepared libraries were isolated and quantified using a Qubit double-stranded DNA (dsDNA) high-sensitivity assay kit (Thermo Fisher, Bangalore, India). Finally, the prepared libraries of all samples were sequenced using the Illumina MiSeq with a 2 × 300 base pair paired end version 3 sequencing kit (San Diego, CA). The sequencing was performed at Biokart India Pvt. Ltd. (Bengaluru, India).

### Data processing.

After sequencing, the binary base call (BCL) file format was converted into FASTQ format. The raw data quality was checked by employing the FastQC (version 0.11.9) and MultiQC (version 1.10.1) tools. Next, elimination of adapters and low-quality reads was completed by using TrimGlore (version 0.6.6). The QIIME (version 1.9.0) workflow, including merging of paired-end reads, chimera exclusion, and OTU abundance calculation, was performed to identify OTUs at the genus level (85). Kraken2 with the UNITE database was used for the molecular identification of fungi (86, 87). Furthermore, data were filtered on the web-based platform MicrobiomeAnalyst to eliminate the low-quality or uninformative features, using as filters minimum count = 4, 20% prevalence, and low variance (interquartile range, 10%) (15). A total of 211 low-abundance features were separated based on prevalence, and a total of 16 low-variance features were removed based on interquartile range. There were 139 features remaining after the data-filtering step.

The fungal community diversity within a product (α-diversity) was estimated by observed (total number of OTUs per sample), Chao1, Fisher, Shannon, and Simpson diversity indices. The different categories of STPs, khaini, moist snuff, qiwam, snus, pan-masala, gul, and zarda, were compared for total features with a one-way analysis of variance (ANOVA) at a *P* value of <0.05. The β-diversity (differences in diversity) between samples was measured by Bray-Curtis distance and plotted using principal-component analysis (PCA) and nonmetric multidimensional scaling (NMDS) (88, 89). The β-diversity between the groups was investigated by permutational multivariate analysis of variance (PERMANOVA), analysis of group similarities (ANOSIM), and homogeneity of group dispersions (PERMDISP) statistical methods using MicrobiomeAnalyst (15). The core microbiome analysis of all STPs was achieved with a sample prevalence cutoff of 20% and relative abundance of 0.01%. The hierarchical clustering of products was generated by applying the Euclidean distance measure along with Ward’s clustering algorithm. The LEfSe algorithm was carried out for the linear discriminant analysis (LDA) to identify fungi as biomarkers, with a *P* value cutoff of 0.05 and log LDA score of 2.0 (90). Furthermore, the correlations among relative abundances of fungal genera were performed by using SparCC (38). A correlation threshold value of >0.3 and statistical significance at a *P* value of <0.05 were considered for plotting the correlation network. The reproducibility of sequencing data was confirmed by using duplicates of snus and zarda samples (S1_dup and Z1_dup).

### FUNGuild analysis.

To investigate the functional potential of STP-associated fungal populations, the overall fungal communities (genus level) were classified into ecological guilds via the FUNGuild database (36). The confidence rankings were “highly probable,” “probable,” and “possible.” The specific functional groups of fungi were divided into three trophic modes, e.g., pathotrophs (gain nutrients from the host), saprotrophs (obtain nutrients by breaking down the dead host cells), and symbiotrophs. The trophic modes were subdivided into fungal functional guilds, and the relative abundances of fungal trophic modes and dominant functional groups between the communities were determined in all STPs. All OTUs that did not match any taxonomic classification were designated “unassigned.”

### Identification of mycotoxin in STPs.

The purification of mycotoxins and their estimation by LC-MS/MS (Eurofins Genomics India Pvt. Ltd., Gurugram, India) was performed as described previously (30). Briefly, the homogenized STP sample (5 to 10 g) was mixed with NaCl (2.5 g) and extracted with a solvent mixture consisting of methanol plus water (50 mL, 80:20 [vol/vol]) and *n*-hexane (25 mL). After that, the mixture was centrifuged at 3,700 rpm for 10 min and the *n*-hexane phase was discarded. The clear extract (1 to 5 mL) was diluted with phosphate-buffered saline and was applied on an immunoaffinity column for the cleanup procedure (immunoaffinity column specific to aflatoxin and ochratoxin antibody-antigen reactions). Analytes were finally eluted with elution solvent (methanol) into collection vials. Next, the vials were vortexed and injected into the LC-MS/MS instrument (Agilent, Santa Clara, CA). The limit of quantification (LOQ) was 0.5 ppb.

### Statistical analysis.

The statistical model ANOVA was used to compare the differences among mean values of a pair of groups belonging to STPs. A *P* value of <0.05 was considered to be statistically significant.

### Data availability.

The metabarcoding data of fungal ITS amplicons was submitted to the NCBI Short Read Archive (SRA) under BioProject accession number PRJNA779591.
